# Influence of genetics and the pre-vaccination blood transcriptome on the variability of antibody levels after vaccination against *Mycoplasma hyopneumoniae* in pigs

**DOI:** 10.1186/s12711-021-00614-5

**Published:** 2021-03-18

**Authors:** Fany Blanc, Tatiana Maroilley, Manuel Revilla, Gaëtan Lemonnier, Jean-Jacques Leplat, Yvon Billon, Laure Ravon, Olivier Bouchez, Jean-Pierre Bidanel, Bertrand Bed’Hom, Marie-Hélène Pinard-van der Laan, Jordi Estellé, Claire Rogel-Gaillard

**Affiliations:** 1 Université Paris‐Saclay, INRAE, AgroParisTech, GABI, 78350 Jouy‐en‐Josas, France; 2 INRAE, GenESI, 17700 Surgères, France; 3 INRAE, GeT-PlaGe, Genotoul, 31326 Castanet-Tolosan, France

## Abstract

**Background:**

The impact of individual genetic and genomic variations on immune responses is an emerging lever investigated in vaccination strategies. In our study, we used genetic and pre-vaccination blood transcriptomic data to study vaccine effectiveness in pigs.

**Results:**

A cohort of 182 Large White pigs was vaccinated against *Mycoplasma hyopneumoniae* (*M. hyo*) at weaning (28 days of age), with a booster 21 days later. Vaccine response was assessed by measuring seric *M. hyo* antibodies (Ab) at 0 (vaccination day), 21 (booster day), 28, 35, and 118 days post-vaccination (dpv). Inter-individual variability of *M. hyo* Ab levels was observed at all time points and the corresponding heritabilities ranged from 0.46 to 0.57. Ab persistence was higher in females than in males. Genome-wide association studies with a 658 K SNP panel revealed two genomic regions associated with variations of *M. hyo* Ab levels at 21 dpv at positions where immunity-related genes have been mapped, *DAB2IP* on chromosome 1, and *ASAP1*, *CYRIB* and *GSDMC* on chromosome 4. We studied covariations of Ab responses with the pre-vaccination blood transcriptome obtained by RNA-Seq for a subset of 82 pigs. Weighted gene correlation network and differential expression analyses between pigs that differed in Ab responses highlighted biological functions that were enriched in heme biosynthesis and platelet activation for low response at 21 dpv, innate antiviral immunity and dendritic cells for high response at 28 and 35 dpv, and cell adhesion and extracellular matrix for high response at 118 dpv. Sparse partial least squares discriminant analysis identified 101 genes that efficiently predicted divergent responders at all time points. We found weak negative correlations of *M. hyo* Ab levels with body weight traits, which revealed a trade-off that needs to be further explored.

**Conclusions:**

We confirmed the influence of the host genetics on vaccine effectiveness to *M. hyo* and provided evidence that the pre-vaccination blood transcriptome co-varies with the Ab response. Our results highlight that both genetic markers and blood biomarkers could be used as potential predictors of vaccine response levels and more studies are required to assess whether they can be exploited in breeding programs.

## Background

Sustainability is one of the main current challenges in livestock farming. In this context, reducing the use of antibiotics and anti-microbials has become a major concern. This can be addressed by considering that animals continually interact with a dynamic and potentially pathogenic ecosystem. Increasing vaccination efficiency is one of the avenues explored to promote sustainable livestock production. To achieve this, it is necessary to better understand the mechanisms that underlie host-pathogens interactions, to develop new vaccines and vaccination strategies, and also to consider genetic improvement of the host response to vaccination and management of its variability [1, 2].

*Mycoplasma hyopneumoniae* (*M. hyo*) bacteria are known to cause enzootic pneumonia, a chronic respiratory disease in pigs, and play a primary role in the porcine respiratory disease complex [3]. *M. hyo* infections cause significant economic losses due to the costs of treatments, reduced animal performance by decreasing growth, and increased mortality from secondary infections [4]. Commercial vaccines are effective in preventing and reducing the severity of lung lesions, and thus in improving daily weight gain and slaughter weight [5–8]. However, they do not reduce the transmission of the pathogen significantly [7, 9]. Therefore, improving vaccination efficacy to *M. hyo* is still an important issue.

Systems vaccinology and vaccinomics, which consist of merging -omics data to comprehensively assess biological systems in response to vaccination, are new approaches that have been proposed in human vaccinology to enhance insights into the evaluation of immune responses and adverse events, and the development of new vaccine candidates [10, 11]. Immunogenetic studies in humans have revealed that single nucleotide polymorphisms (SNPs) in human leukocyte antigen class I and class II, cytokine, cytokine receptor, and innate immune response (e.g., toll-like receptor) genes may partly account for the inter-individual variability of the immune response to various vaccinations (measles and rubella [1, 12–16], hepatitis B [1, 17–19], influenza [20], smallpox [21] or *Bacillus anthracis* [22]). In humans, host genetics has been reported to influence the persistence of specific antibodies (Ab) throughout life, after vaccination against tetanus toxoid, influenza B virus, and capsular group C meningococcal during childhood [23]. In livestock animals, the role of host genetics in the variability of Ab response to vaccines has been documented for respiratory syncytial virus vaccine in bovine [24] and for Newcastle disease virus vaccine in chicken [25]. In pigs, across-breed variability of response levels to vaccine against Aujeszky’s virus disease has been reported [26]. Within-population variability has been shown for Ab response to vaccines against influenza [27] and porcine reproductive and respiratory syndrome [28] viruses, and tetanus bacteria [29], and to the bacterial antigens K88ab, K88ac, and O149 [30]. We and others have also reported individual variability of serum *M. hyo*-specific Ab induced after vaccination in various populations [31, 32].

Blood is an accessible fluid that reflects the status of the immune system [33]. Thus, in vaccine research, it serves as a surrogate tissue to identify potential markers of vaccine-induced responses. In swine, we have shown that the peripheral blood transcriptome reflects variations in innate and adaptive immunity traits [34]. In humans, blood transcriptomic profiling has identified both early innate [35, 36] and concomitant adaptive humoral immunity gene signatures after vaccination [37]. In particular, Li et al*.* [38] defined early transcriptional signatures of Ab responses that were derived from a systems biology study. Such studies have also been recently conducted in sheep [39, 40] and pigs [41–43], and have provided insights into the pathways involved in vaccination responses. However, to date, the underlying determinants of immune capacity that are involved in vaccination responses have not been identified.

In this study, our aim was to characterize individual variability of vaccine response to *M. hyo* in pigs and to identify genetic parameters and baseline blood transcriptomic profiles that could predispose to effective response to vaccination and predict associated Ab response levels. Thus, we combined data from high-density genotyping, the transcriptome of blood collected before vaccination, and *M. hyo*-specific Ab levels at various time points after vaccination.

## Methods

### Animal design, zootechnical traits, and sampling

**Fig. 1:**
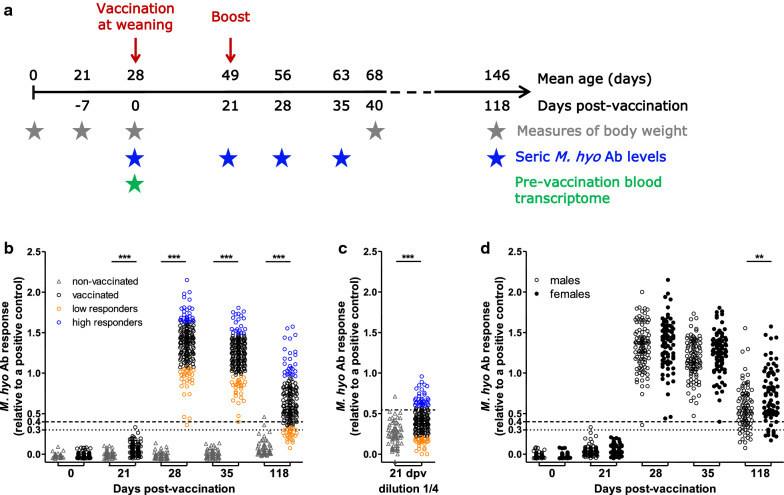
Individual variability of *M. hyo* Ab response in pigs’ sera after vaccination. **a** Vaccination, blood sampling protocol and associated data. The time points for body weight measures and blood samples are represented by stars. **b** Levels of *M. hyo* Ab in 1/40 diluted sera from vaccinated and non-vaccinated animals at 0 (day of vaccination), 21 (day of booster), 28, 35, and 118 days post vaccination (dpv). Dotted lines represent thresholds of the assay given by the provider. Statistical analyses: unpaired t test between controls and vaccinated animals (***p < 0.001). **c** Levels of *M. hyo* Ab in 1/4 diluted sera from vaccinated and control animals at 21 dpv. Dotted lines represent thresholds of detection (mean + 2 SD of the controls). Statistical analyses: unpaired t test between controls and vaccinated animals (***p < 0.001). **d** Levels of *M. hyo* Ab in 1/40 diluted sera from vaccinated males and females at 0, 21, 28, 35, and 118 dpv. Dotted lines represent the thresholds of the assay given by the provider. Statistical analyses: data were fitted using a mixed linear regression model with sex, age at weaning and batch as fixed effects, and litter as random effect; and effect of sex was evaluated by ANOVA (**p < 0.01)

In total, 48 litters of Large White pigs were produced in five batches and raised without antibiotic treatment on the GenESI, INRAE, Pig Innovative Breeding Experimental Facility (https://doi.org/10.15454/1.5572415481185847E12). Sows (n = 47) were inseminated with boar semen (n = 48) that was selected to maximize genetic variability, with one sow inseminated for two parities. From each litter, three to four piglets of each sex were chosen based on their weight at 21 days, taking care to represent average litter weight piglets and avoid animals with a too low weight. This resulted in a set of 278 piglets (145 uncastrated males and 133 females) that defined the experimental population. At random four to five piglets per litter were vaccinated against *M. hyo* (Stellamune, Pfeizer), and the remaining piglets (one or two animals per litter) were not vaccinated. In total, the experimental population included 203 vaccinated animals and 75 control non-vaccinated animals. The experimental design with the associated measures at different time points is summarized in Fig. 1a. A first injection was administered at 0 dpv, corresponding to the day of weaning (at 28 days of age on average, from 24 to 31 days). A booster vaccination injection was given at 21 dpv. A small subset of animals was removed from the initial population due to farming problems that led to morbidity and premature death (n = 13 vaccinated and 11 controls) or to no response to vaccination during the experiment (n = 4). The latter four animals were not included because we could not conclude whether they were non-responders or whether the vaccination injection had failed.

The animals were raised under standard conditions in pens of 20 to 30 animals during the post-weaning period (from around 28 to 68 days of age) and in pens of 10 to 12 animals during the growth period (from around 68 to 146 days of age). They all received the same standard commercial diets. The final dataset comprised 186 piglets vaccinated against *M. hyo* and 64 control non-vaccinated piglets, of which 170 and 61, respectively, were monitored until 118 dpv. The final individual dataset is in Additional file 1: Table S1. Body weights (BW) were determined at different time points: at birth, 7 days before weaning (dbw) at around 21 days of age, at weaning at around 28 days of age (corresponding to 0 dpv), at the end of the post-weaning period (corresponding to 40 dpv) at around 68 days of age, and at the end of the experiment, before slaughter (corresponding to 118 dpv) at around 146 days of age. Average daily gain (ADG) was calculated from 0 to 40 dpv (ADG 0–40 dpv) and from 40 to 118 dpv (ADG 40–118 dpv).

Peripheral blood was sampled from the jugular vein at different time points using dry tubes (Becton Dickinson) for serum preparation (0, 21, 28, 35, and 118 dpv), EDTA-coated tubes (Becton Dickinson) for DNA extraction (0 dpv), and Tempus tubes (Thermo Fisher) for RNA extraction (0 dpv). Blood samples were stored at − 20 °C (EDTA tubes) or at − 80 °C (Tempus tubes) prior to DNA or RNA extraction, respectively. Peripheral blood was also sampled from sows on dry tubes during the week before parturition to check that they were seronegative for *M. hyo*-specific Ab.

### Measurement of *M. hyo*-specific Ab levels and classification of animals based on Ab response

The levels of *M. hyo*-specific Ab were measured by using a commercial ELISA test (IDEXX *M. hyo* Ab test, IDEXX Europe B.V., The Netherlands) and running duplicates of sera diluted 1/40 for all time points (0, 21, 28, 35, and 118 dpv). At 21 dpv, *M. hyo*-specific Ab were also measured with sera diluted 1/4. Ab levels were calculated by dividing the absorbance of the samples (S, corrected by subtraction of the mean negative control absorbance) by the mean absorbance of the positive control (P, corrected by subtraction of the mean negative control absorbance), which resulted in an S/P value following the IDEXX procedure. Given the thresholds provided by IDEXX, samples with S/P values higher than 0.4, within the range from 0.3 to 0.4, or lower than 0.3, were assessed as positive, suspect, or negative for *M. hyo* Ab, respectively.

To carry out differential analyses between animals with high and low vaccine responses, we identified extreme groups for *M. hyo*-specific Ab levels at 21, 28, 35, and 118 dpv, using the S/P values obtained for the whole pig population at each time point (n = 186 at 21, 28, and 35 dpv and n = 170 at 118 dpv). High (low) responders corresponded to pigs with an Ab response higher (lower) than the mean plus (minus) one standard deviation (SD) (n = 34, 30, 22, and 27 for high responders, colored in blue in Fig. 1a, b, and n = 32, 29, 28, and 25 for low responders, colored in orange in Fig. 1a, b at 21, 28, 35, and 118 dpv, respectively). Positive (pos) and negative (neg) animals were defined at 118 dpv based on the thresholds of the Ab test given by the manufacturer (neg: S/P value < 0.3, n = 19, pos: S/P value > 0.4, n = 136). For Ab response at 21 dpv, the mean plus 2 SD of the values obtained for non-vaccinated control animals were used to determine detection thresholds (0.07 at 1/40 dilution and 0.545 at 1/4 dilution). Animals for which values were below vs above these thresholds at both dilutions were classified as negative (n = 134) vs positive (n = 32). Animal groups at each time point are in Additional file 1: Table S1 and descriptive statistics of the groups are in Additional file 1: Table S2.

### Effect of zootechnical parameters on vaccination response and of vaccination on body weight

Unless otherwise notified, all data analyses were conducted with the R software (v3.6.1) [44]. The effects of different zootechnical parameters on *M. hyo* Ab responses were evaluated with a linear mixed model using the lmer function of the lme4 (v1.1-21) R package [45], with sex (two levels) and batch (five levels) as fixed effects, age at weaning (between 24 and 31 days) as a linear covariate, and litter (48 levels) as a random effect. To evaluate the effect of vaccination on weight traits, vaccine groups (vaccinated vs non-vaccinated; or high vs low; or negative vs positive) were also included in the model as fixed effects. *P*-values for fixed effects were obtained using the lmerTest package (v3.1-1) [46] by type III ANOVA tables, with Satterthwaite’s approximation to degrees of freedom using the ANOVA function, while *p*-values for random effects were calculated by the likelihood ratio test using the rand function. Pairwise comparisons with Tukey’s adjustment were performed to assess the differences between batches, using the emmeans function of the emmeans package (v1.5.2-1). A significance level of 0.05 was applied. Pearson’s correlation matrices were built with the corrplot R package (v0.84). Correlations of the repeated measures of Ab response levels at the different time points post-vaccination were also calculated using a linear mixed model with dpv as fixed effect, animal as random effect, and an autoregressive correlation structure of order 1 (corCAR1 function) across dpv, using the nlme package (v3.1-141). Principal component analyses (PCA) were performed with the FactoMineR R package (v2.1) [47] and the results were visualized based on the factoextra R package (v1.0.6).

### Estimation of heritabilities

Genetic parameters (heritabilities and genetic correlations) were estimated using the restricted maximum likelihood methodology applied to a multivariate mixed linear model with the same fixed effects and covariates (sex, batch and age at weaning) as above, and the additive genetic value of each animal and a residual error as random effects, which is the so-called animal model, where the vector of additive genetic values is assumed to be proportional to the numerator relationship matrix built from pedigrees. Computations were performed using the VCE6 software [48].

### Genome-wide association studies (GWAS)

Vaccinated pigs (n = 186) were genotyped with a high-density SNP panel (Affymetrix AXIOM PIG HD, 658 K SNP). Genotyping of four pigs (2.1%) did not pass various internal quality controls (QC) that identify poor quality samples using the Axiom Suite [Dish QC (n = 2) and sample QC call rate test (n = 2)]. Among the genotypes for the 182 remaining animals that passed plate QC (see Additional file 1: Table S1), only annotated autosomal SNPs were kept for analysis (n = 598,138; Axiom_PigHD_v1.na35.r4.a2.annot.csv annotation file). The check.marker function of the GenABEL package (v1.8-0) in R was applied to filter out SNPs with a minor allele frequency lower than 5% (89,175 SNPs excluded), a call rate lower than 95% (58,869 SNPs excluded), or SNPs that departed from Hardy–Weinberg equilibrium (FDR lower than 0.1; 41,149 SNPs excluded). After applying these QC measures, 425,567 SNPs on 182 animals were retained for GWAS. Overall, the QC analysis of the genotyping data did not identify outlier animals and the genomic kinship coefficients between individuals were consistent with the known pedigrees. GWAS were performed with the RepeatABEL R package (v1.1) [49], by using a linear mixed model with batch and sex as fixed effects, age at weaning as a linear covariate, and litter and the genomic kinship matrix (built with the ibs function) as random effects. A significance level of 0.05 and a suggestive significance level of 0.1 were applied. Finally, the detected associated regions were mapped to the pig genome assembly available at the UCSC Genome Browser on Feb. 2017 (Sscrofa11.1).

### Blood transcriptome by RNA-Seq

RNA from blood samples that were collected in Tempus tubes prior vaccination at 0 dpv was extracted from a subset of 85 vaccinated pigs (see Additional file 1: Table S1), using the Norgen Preserved Blood RNA Purification Kit I (adapted to blood samples collected in Tempus tubes) according to the manufacturer’s instructions. Concentration of the extracted RNA was measured with a NanoDrop 2000 spectrophotometer (89.4 ± 26.0 µg were obtained per sample) and RNA integrity was assessed by an Agilent 2100 Bioanalyzer, using the eukaryote total RNA 6000 Nano Kit (RIN obtained were 8.0 ± 0.6, ranging from 7.1 to 9.3).

Libraries were prepared from 1 µg of total RNA with the Illumina TruSeq stranded total RNA with Ribo-Zero Globin sample preparation kit. Following the manufacturer’s protocol, ribosomal and globin RNA were removed by depletion and the remaining coding and non-coding RNA was used as input for library preparation. RNA was fragmented using Illumina’s fragmentation enzyme mix (Elute, Prime, Fragment Mix) for 8 min at 94 °C. For synthesis of the first strand, 1 µL of SuperScript II Reverse Transcriptase was mixed with 9 µL of Illumina’s First Strand Mix with actinomycin D, and then 8 µL of this mix were added to the fragmented RNA, and PCR was carried out in a thermocycler that was programmed as indicated in the TruseqRNA protocol. To generate double strand (ds) cDNA, 20 µL of Illumina’s Second Strand mix were mixed with the first strand cDNA and incubated for one hour at 16 °C. Then, 90 µL of AMPure XP beads were used to purify the ds cDNA that was eluted in 15 µL of Illumina’s resuspension buffer. The ds cDNA was end-repaired, adenylated, and then Illumina adapters were added, as indicated in the TruSeq stranded mRNA protocol. The prepared libraries were quality-checked with the high sensitivity D1000 screen Tape (Agilent Tape Station 2200), quantified with Qubit (ThermoFisher), and 12-plex pooled. The pooled libraries were quantified with the Qubit dsDNA HS (High Sensitivity) Assay kit and sequenced on the GeT-PlaGe core facility (INRAE, https://doi.org/10.15454/1.5572370921303193E12) on the Illumina Hiseq3000 sequencer with a 150PE module, with each pool run in two Hiseq3000 lanes.

The reads were mapped to the pig genome assembly Sscrofa11.1 (Ensembl v90 release) by using TopHat (v2.1.0), and the read counts for each gene were obtained by using htseq_count (v0.6.1.p1). Overall, the RNA-Seq data provided a sufficient number of reads per sample (mean number of reads larger than 60 × 10^6^). Three animals with a total number of reads smaller than 20 million were excluded. Thus, the final dataset for the transcriptome analysis included 82 vaccinated pigs, of which 15, 14, 11, and 11 were high responders at 21, 28, 35, and 118 dpv, respectively, 15, 23, 19, and 13 were low responders at 21, 28, 35, and 118 dpv, respectively, and 55 and 10 were negative, and 14 and 64 were positive at 21 and 118 dpv, respectively. These pigs were representative of the corresponding groups from the whole population, showing equivalent means of Ab levels (see Additional file 1: Table S2).

### Weighted gene correlation network analysis (WGCNA) and correlation with vaccine-induced Ab responses

WGCNA [50] was conducted in R to find clusters of highly correlated genes within the RNA-Seq dataset. Read counts per gene were filtered by retaining only the genes with more than 1 count per million and more than 10 reads for at least one third of the animals. Read counts were then normalized with the calcNormFactors function implemented in the edgeR package (v3.26.6). Finally, limma’s voom function (v3.40.6) was used to fit a generalized linear regression model to correct the data with sex and batch as fixed effects, and age at weaning as a linear covariate. Samples were then clustered based on their Euclidean distance (hclust function of fastcluster package v1.1.25) and six outliers were removed (SPH_034, SPH_058, SPH_084, SPH_193, SPH_195, and SPH_417). Thus, 76 samples were used for analysis. Block-wise network construction and module detection were carried out with the WGCNA package (v1.68) in two blocks using the blockwiseModules function with a threshold power of 6, a height of 0.25, a deep split level of 2, a reassign threshold of 0.2, and a minimum module size of 30. The eigenmodules (essentially the first principal component of the modules, which can be used as a synthesis often referred to as a “signature” of the module gene expression) were then correlated with Ab responses. Modules that were correlated (Pearson correlation) with the Ab responses with a *p*-value lower than 0.05 were considered significantly correlated.

### RNA-Seq-based differential expression analyses of blood genes between pigs that differed in Ab responses to vaccine

Differential expression (DE) analyses were conducted using the edgeR package (v3.26.6) in R. Read counts were filtered, normalized, and corrected for sex, age at weaning and batch effects as described for the WGCNA analysis. Then, likelihood ratio tests were performed to test, for each gene, the differential expression between extreme groups. The *p*-values were adjusted using the false discovery rate (FDR) method [51] and a significance level of 0.05 and a suggestive significance level of 0.1 were applied.

### Feature set enrichment analyses (FSEA)

FSEA were performed with the R package tmod (v0.40) [52]. Hypergeometric tests were used to determine the enrichment of each WGCNA module that was significantly correlated with Ab responses (foreground set) in the total RNA-Seq gene set (background set). The coincident extreme ranks in numerical observations (CERNO) method was used to analyze the feature set enrichment using lists of genes obtained from the DE analyses and ranked by the absolute logarithm of fold change (logFC). To interpret the feature set enrichments, we used gene collections consisting of the blood transcriptomic modules (BTM) and signatures annotated by Li et al*.* [38], which were adapted by replacing human genes with their corresponding genes in pigs by Matthijs et al*.* [43], and FSEA were visualized with tmodPanelPlot.

### Sparse partial least square-discriminant analysis (sPLS-DA) and PLS-DA

Sparse partial least square-discriminant analyses (sPLS-DA) [53] were performed with the mixOmics R package (v6.10.8) to identify genes that were expressed in blood before vaccination and that were the most discriminative features of the response to vaccination. Read counts were filtered, normalized, and corrected for sex, age at weaning, and batch effects, as described for the WGCNA analysis. The classification performance (error rate) was estimated with the function tune.splsda of the mixOmics R package. The tuning was first performed one component at a time, with a maximum of ncomp = 3 and with 5 to 95 (step of 5) genes to test per component, and fivefold cross-validation repeated 100 times. In all analyses, one component was sufficient to provide the lower error rates. The optimal number of genes to select was then refined by performing another tuning in a more restricted range of genes to test, based on the error rate of the obtained profiles and with a maximum of 25 genes (step of 1), and fivefold cross-validation repeated 100 times. The final models included one component and the determined number of genes to be selected that led to the best performance for predicting the classification of animals in high vs low Ab responders at 21, 28, 35, and 118 dpv and positive vs negative Ab responders at 21 and 118 dpv. The set of predictive genes was defined by combining all these genes, and PLS-DA were finally performed to evaluate the predictive capacities of this final set of 101 genes.

## Results

### Individual variability of specific antibody response after *M. hyo* vaccination

All individual data and metadata are in Additional file 1: Table S1. The *M. hyo*-specific Ab response was monitored by measuring seric *M. hyo*-specific IgG levels at four time points, corresponding to three physiological steps of the humoral antibody response: early response after one injection of vaccine at 21 dpv (49 days of age), maximum intensity of the response after the booster vaccination at 28 and 35 dpv (56 and 63 days of age, respectively), and persistence of the Ab response until slaughter at 118 dpv (146 days of age) (Fig. 1a). At 21 dpv, *M. hyo* Ab levels were significantly higher in vaccinated than in control animals but only one animal reached the threshold that indicated a *M. hyo* Ab response (Fig. 1b). Thus, at this early time point, we ran the assay with more concentrated sera (1/4 dilution, Fig. 1c) to better assess the variability among animals. At this dilution, *M. hyo* Ab levels were also significantly higher in vaccinated than in control animals. *M. hyo* Ab response reached maximum intensity at 28 dpv for 79% of animals and at 35 dpv for the other animals. At 118 dpv, *M. hyo* Ab levels remained above the threshold for 80% of the vaccinated pigs, which indicated a persisting humoral response for these animals. Among the other animals, 8.2 and 11.8% had suspect or negative *M. hyo* Ab levels, respectively.

We observed a high individual variability of the *M. hyo* Ab levels at each time point (Fig. 1b and Table 1). The coefficients of variation (CV) were equal to 46% at early (21 dpv, serum dilution 1/4) and late time points (118 dpv), and 20 and 21% at the maximum response intensity (28 and 35 dpv). At 21 dpv, the mean values of seric Ab levels with the 1/40 dilution were very low compared to the other values, with a CV that reached 151% (Table 1). In the next steps, for that time point, we used only the Ab levels measured with the 1/4 dilution, which provided a better range of individual observations.

**Table 1:** Descriptive statistics and heritability estimates of *M. hyo* Ab vaccine response at different days post-vaccination (dpv)

Type of Ab response	dpv	Serum dilution	S/P values^a^			h^2^ (SE)
			Mean	SD	CV (%)	
Early (before booster)	21	1/40	0.041	0.06	151	NC
		1/4	0.403	0.19	46	0.57 (0.15)
Maximum (post booster)	28	1/40	1.349	0.28	21	0.46 (0.11)
	35	1/40	1.215	0.25	20	0.52 (0.11)
Persistence (before slaughtering)	118	1/40	0.645	0.30	46	0.52 (0.11)

*NC* not calculated

^a^Ab levels are expressed relatively to a positive control (S/P values, see Methods)

We assessed the effect of known factors that included sex, age at weaning, batch, and litter on *M. hyo* Ab levels. Batch had a significant (*p*-values < 0.05) effect at 28 and 118 dpv, litter at 21 dpv, and sex at 118 dpv (see Additional file 1: Table S3). Ab levels of animals from B_1602 were lower than those from B_1611 at 28 dpv and lower than those from B_1604 at 118 dpv (*p*-values < 0.05). Please note that females exhibited 30% higher levels of specific Ab in response to vaccination at 118 dpv than males (see Additional file 1: Table S3) and Fig. 1d.

### Correlation between *M. hyo* Ab levels at different time points

**Fig. 2:**
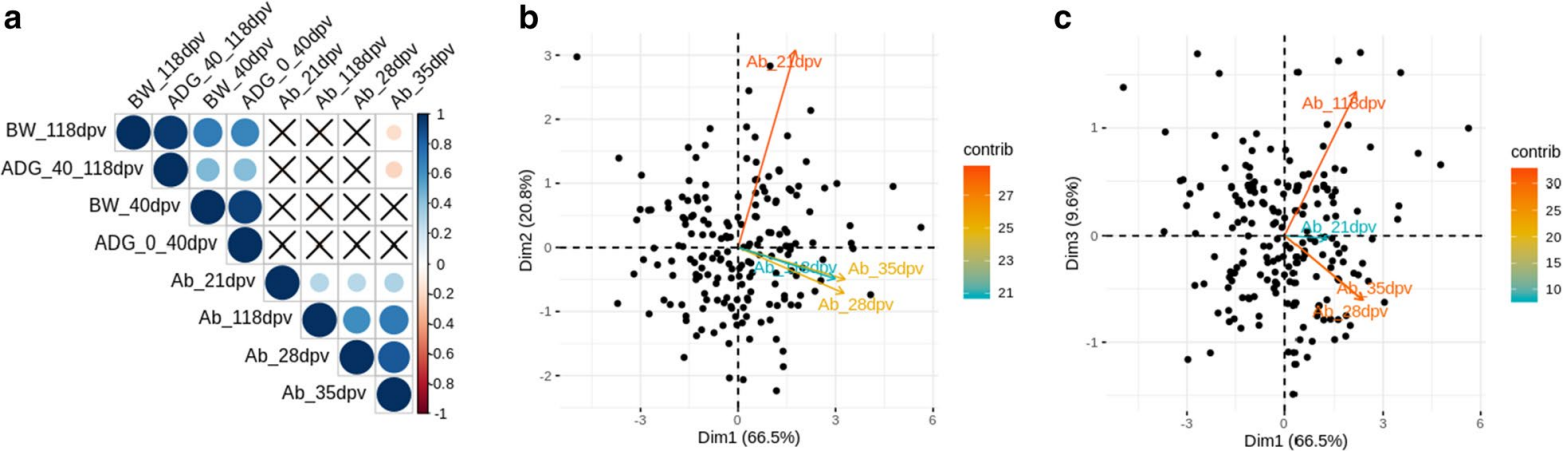
Correlations of *M. hyo* Ab levels with production traits. **a** Correlation matrix (Pearson correlation coefficients) of *M. hyo* Ab levels in sera at 21, 28, 35, and 118 dpv; BW at 40 and 118 dpv; and ADG from 0 to 40 dpv and 40 to 118 dpv. Color intensity and size of the circle are proportional to the correlation coefficients. Non-significant (*p* > 0.05) correlations are crossed out. **b**, **c** Principal component analysis (PCA) of *M. hyo* Ab levels in sera at 21, 28, 35, and 118 dpv for components **b** 1–2 and **c** 1–3

*M. hyo* Ab levels at different time points were positively correlated with one another (Fig. 2a). Ab levels at 28 and 35 dpv were highly correlated with each other (r = 0.84) and were correlated with Ab levels at 118 dpv (r = 0.62 and 0.71, respectively). Ab levels at 21 dpv were moderately correlated with later time point responses (r ranging from 0.27 to 0.3). Since *M. hyo* Ab levels at different time points can be considered as repeated measurements, temporal correlations were evaluated and were found to be positive, ranging from 0.46 to 0.68 (see Additional file 1: Table S4). In a principal component analysis (PCA) of *M. hyo* Ab levels at all time points, animals were distributed along the first component mostly by their Ab responses at 28, 35 and, 118 dpv and along the second component by their Ab responses at 21 dpv (Fig. 2b). The two first components explained 66.5 and 20% of the variance, respectively. The third component separated animals by their Ab responses at 28 or 35 dpv vs 118 dpv (Fig. 2c).

### Comparison of vaccinated and non-vaccinated groups for body weight

We assessed the effect of vaccination on BW after 40 and 118 dpv and on ADG during the post-weaning (0–40 dpv) and growing (40–118 dpv) periods. We applied a mixed linear model with vaccine group (vaccinated or non-vaccinated), sex, and batch as fixed effects, age at weaning and BW at 0 or 40 dpv as linear covariates, and litter as a random effect. Sex had a significant (*p*-values < 0.005) effect on BW at 118 dpv and on ADG at 40–118 dpv (with higher values for males). Batch had a significant effect on BW at 40 dpv and on ADG at 0–40 dpv. BW at 0 dpv had also a significant effect on all BW measurements, with a higher BW at 0 dpv and a higher BW or ADG at older ages. Vaccination group had no significant effect on BW when the two groups of vaccinated and non-vaccinated pigs were compared (see Additional file 1: Table S5).

### Relationships between *M. hyo* Ab response and body weight in the vaccinated population

To evaluate the relationships between BW and *M. hyo* Ab levels after vaccination, we performed a correlation analysis (Fig. 2a). BW measurements at different time points were positively and significantly (*p*-values < 0.05) correlated throughout the animals’ lifetime. The ADG during the post-weaning period (0–40 dpv) and during the growing period (40–118 dpv) were also positively correlated (Fig. 2a). As shown in Fig. 2a, Ab levels at 35 dpv and BW at 118 dpv or ADG during the growing period (from 40 to 118 dpv) were slightly but significantly negatively correlated (r = -0.18 and-0.22, respectively). However, no significant correlations were found between the Ab levels at other time points and BW using the whole vaccinated population (Fig. 2a).

Then, we compared BW measurements between animals with high vs low or negative vs positive Ab response by including the corresponding vaccine group as a fixed effect in the model. Interestingly, this approach revealed that high Ab responders at 28 dpv had 6.4 and 7.3% lower BW at 40 and 118 dpv, respectively, and had 9.7% and 8.3% lower ADG from 0 to 40 dpv and from 40 to 118 dpv, respectively (Table 2, *p* < 0.05). In addition, animals with a positive (vs negative) Ab level at 21 dpv had 5.3% lower BW at 40 dpv and 8.1% lower ADG from 0 to 40 dpv (*p* = 0.007). Thus, overall, these results show a slight but significantly reduced BW and ADG for the higher Ab vaccine responders that could be detected as soon as 40 dpv and persisted until slaughter at 118 dpv.

**Table 2:** Estimates of differences in body weight (BW, kg) at 40 and 118 dpv and in average daily gain (ADG, kg/day) from 0 to 40 dpv and from 40 to 118 dpv between pigs with high vs low or positive vs negative *M. hyo* Ab response

Groups of contrasted Ab responses to vaccination		BW at 40 dpv	ADG 0–40 dpv	BW at 118 dpv	ADG at 40–118 dpv
dpv	Classification				
21	High vs low	0.102	0.099	0.088	0.194
	Positive vs negative	*0.007*^a^	*0.007*^a^	0.054	0.191
28	High vs low	*0.025*^b^	*0.025*^b^	*0.019*^b^	*0.029*^b^
35	High vs low	0.444	0.443	0.113	0.128
118	High vs low	0.259	0.262	0.143	0.182
	Positive vs negative	0.833	0.841	0.418	0.310

^a^negative > positive; ^b^low > high

High vs low and positive vs negative animals were defined as described in the Methods section. Details on the assignment of animals to groups at each time point are provided in Additional file 1: Table S1. BW was fitted using a linear mixed model with sex, batch and groups of contrasted responses to vaccination at each time point (high vs low or positive vs negative) as fixed effects, age at weaning and BW at 0 dpv (weaning) as linear covariates, and litter as random effect. Classification *p*-values of the effect of contrasted groups of vaccine responders at each time point are in this table (significant *p*-values are in italics)

### Genetics of vaccine Ab response

Estimates of the heritability of *M. hyo* Ab response levels were within the same range at all time points (0.46 to 0.57), with a tendency to be slightly higher at the earliest time point (Table 1 and Additional file 1: Table S4). Estimates of heritabilities and phenotypic and genetic correlations between Ab responses to *M. hyo* vaccination at the different time points are summarized in Additional file 1: Table S6. Estimates of genetic correlations of Ab levels at 21 dpv with Ab levels at the three later time points (92 to 98%) were very high. The lowest correlations were detected between Ab levels at 28 and 35, and Ab levels at 118 dpv (0.66 and 0.78, respectively). Estimates of phenotypic correlations were all lower than the corresponding estimates of genetic correlations, especially for correlations of Ab response at 21 dpv with later time points (see Additional file 1: Table S6).

**Fig. 3:**
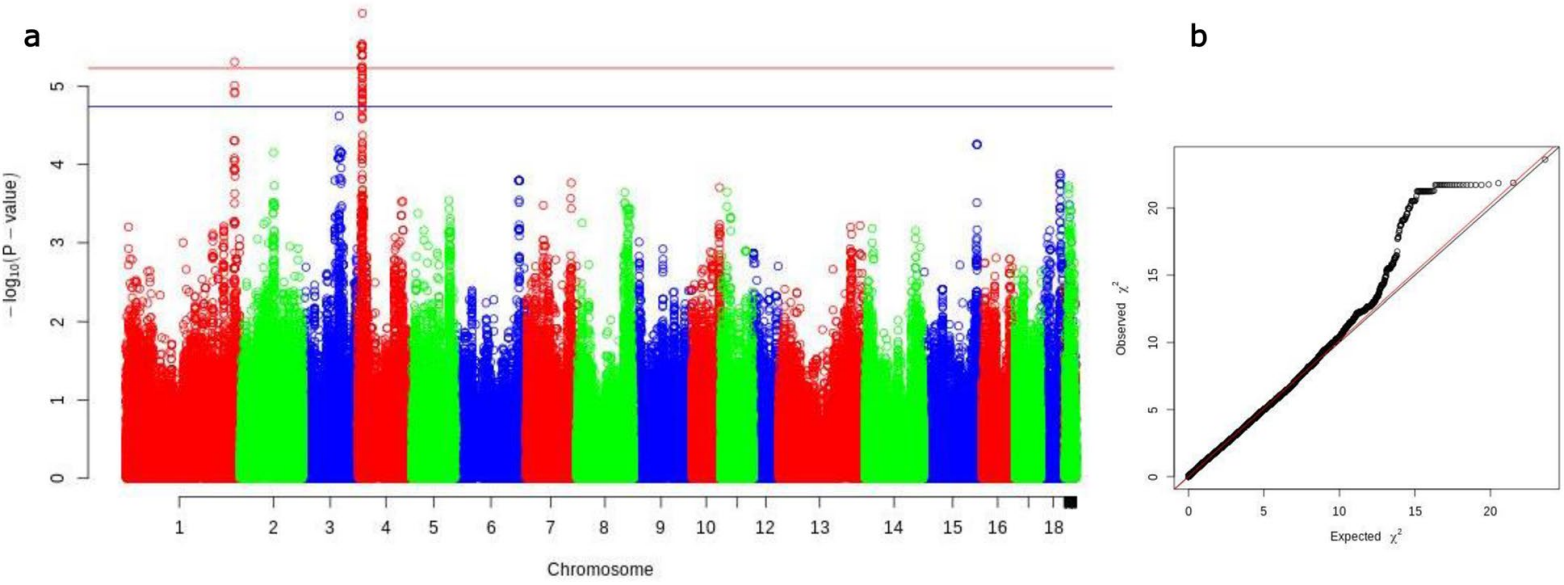
Genome-wide association for *M. hyo* Ab levels at 21 dpv. *M. hyo* Ab levels were assessed in sera diluted 1/4. **a** Manhattan plot based on -log 10 (*p*-value) from GWAS and imputation analysis against chromosome position annotation of the swine genome assembly 11.1 (*Sscrofa11.1*). The blue line indicates suggestive association threshold (FDR = 0.1) and the red line indicates genome-wide significant threshold (FDR = 0.05). **b** QQ plot showing the expected distribution of association statistical tests (X-axis) across the SNPs compared to the observed values (Y-axis)

By performing GWAS for each of the Ab phenotypes, we were able to identify two genomic regions that were associated with *M. hyo* Ab levels at 21 dpv with sera diluted 1/4 (Fig. 3 and Additional file 1: Table S7). The QTL on *Sus scrofa* chromosome 1 (SSC1) SSC1:261,713,894–261,843,495 (129,601 bp long) included three SNPs with FDR < 0.1 and one SNP (AX-116155504) with FDR = 0.047. The QTL on SSC4, between SSC4:10,201,158 and SSC4:11,076,588 (875,430 bp long), included 24 SNPs with a FDR < 0.1 and 51 SNPs with a FDR < 0.05, with AX-116223305 being the most strongly associated SNP (FDR = 0.04). The annotated genes that mapped to these QTL are *DAB2IP* for the QTL on SSC1 and *ASAP1*, *CYRIB,* and *GSDMC* for the QTL on SSC4.

### Correlations of *M. hyo* Ab levels with co-expressed genes in pre-vaccination blood

**Fig. 4:**
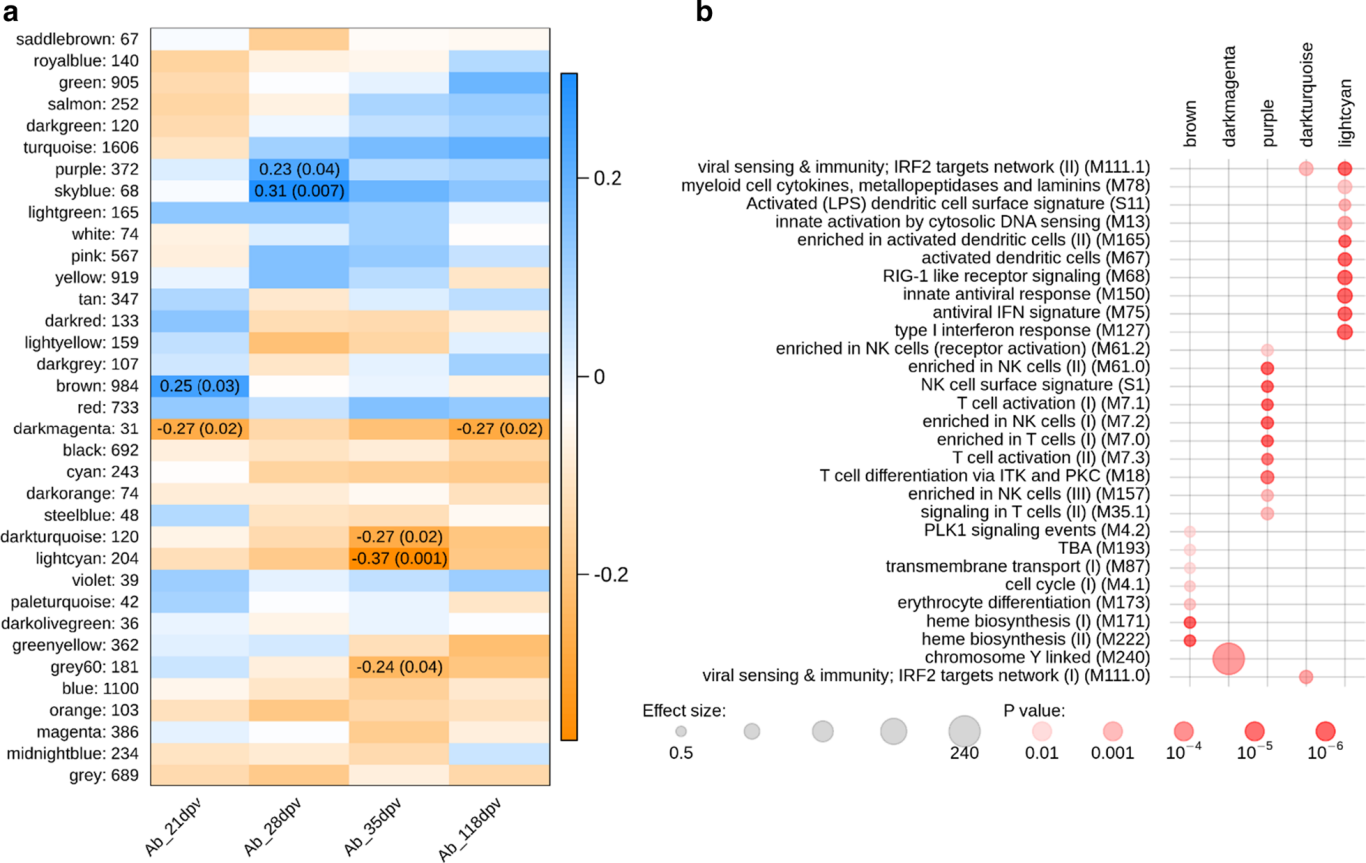
Weighted gene co-expression network analysis (WGCNA) of modules and *M. hyo-*specific Ab response relationships. **a** Modules extracted by co-expression analyses in blood RNA-Seq dataset (at 0 dpv) are in rows labelled with a color; the number of genes contained in the given module is indicated after the color. Genes that were not assigned to a specific module (n = 689) are grouped in the last module labelled in grey color. The heatmaps are color-coded according to Pearson’s correlation coefficient between the given module eigengene and the *M. hyo* Ab levels at 21, 28, 35, and 118 dpv (from positive in blue to negative in orange). Correlation coefficients along with their *p*-value in parenthesis are mentioned when the *p*-value < 0.05. **b** Feature set enrichment analyses (FSEA) of modules correlated with *M. hyo-*specific Ab responses. Enrichment was calculated with hypergeometric tests for each WGCNA module (foreground set) among the total RNA-Seq gene set (background set). Only the WGCNA modules that showed a significant enrichment (hypergeometric test adjusted *p-*value < 0.01) in any of the BTM were included. The strength of the *p*-value is illustrated by a gradient of red. The effect size corresponds to the enrichment calculated as (b/n)/(B/N) where b and B are the numbers of genes from the given module in the fg and bg sets, respectively; n and N, the sizes of the fg and bg sets, respectively. (TBA = to be annotated)

We performed transcription profiling in blood collected before vaccination for a subset of 82 pigs, by RNA-Seq and extracted modules of co-expressed genes using WGCNA. We identified 34 modules that included 31 to 1606 genes. Seven modules had at least one significant correlation with *M. hyo* Ab levels at 21 (two modules), 28 (two modules), 35 (three modules), and 118 dpv (one module) (Fig. 4a, p < 0.05). Only the dark magenta module was significantly correlated with *M. hyo* Ab levels at two different time points (21 and 118 dpv), the six other modules were correlated at only one time point. All significant correlations were low to moderate, with absolute values ranging from 0.23 to 0.37. However, each of these seven modules was correlated with *M. hyo* Ab levels in the same orientation at each time point: positive correlations for purple, sky blue, and brown modules; negative correlations for dark magenta, dark turquoise, light cyan, and grey60 modules (Fig. 4a). The brown module was positively correlated with vaccine response at 21 dpv (r = 0.25), whereas the dark magenta module was negatively correlated (r = − 0.27). Modules that were positively correlated with the *M. hyo* Ab levels at 28 dpv were the purple (r = 0.23) and sky blue (r = 0.31) modules. The three modules that were negatively correlated with the *M. hyo* Ab levels at 35 dpv were the dark turquoise (r = − 0.27), light cyan (r = − 0.37), and grey60 (r = − 0.24) modules. Only the dark magenta module had a significant negative correlation with the *M. hyo* Ab levels at 118 dpv (r = − 0.27).

Five of the seven WGCNA modules that were significantly correlated with Ab levels were enriched in blood transcriptomic modules (BTM) defined by Li et al. [38] (Fig. 4b and Additional file 1: Table S8). The dark magenta module was enriched in the BTM annotated “chromosome Y linked”. The brown module was enriched in the BTM annotated for heme biosynthesis (M171 and M222), cell cycle (M4.1 and M4.2) and dendritic cell/antigen presentation (M87). The purple module had functions enriched in BTM annotated for cell migration (M45 and M91), natural killer cell enrichment and activation (M7.2, MM61.0, M61.2, M157 and signature S1), and T cell enrichment and activation (M7.0, M7.1, M7.3, M18, M35.0 and M35.1). The dark turquoise and light cyan modules had annotated functions in innate antiviral immunity (M13, M68, M75, M111.0, M111.1, M127 and M150), inflammation/immune response (M24, M37.0, M78 and M112.0), and activation of dendritic cells (MS11, M67 and M165).

### Identification of differentially expressed genes in blood before vaccination between pigs with divergent Ab responses to the *M. hyo* vaccine

Differential expression analyses compared groups of high vs low extreme Ab responders at 21, 28, 35, and 118 dpv, and groups of positive vs negative Ab responders at 21 and 118 dpv. We identified four differentially expressed genes at 21 dpv between positive and negative Ab responders and one differentially expressed gene at 28 dpv between high and low responders (Table 3). A set of 52 genes were differentially expressed between the two groups of responders at 118 dpv at FDR < 0.1, including a subset of seven genes with FDR < 0.05.

**Table 3:** Differentially expressed genes in blood before vaccination between groups of animals with divergent Ab responses to *M. hyo* vaccination at different days post-vaccination (dpv)

Type of Ab response	dpv	Classification	Number of differentially expressed genes	Differentially expressed genes with HUGO gene nomenclature committee symbol	
				Higher expression in "high" or "pos"	Higher expression in "low" or "neg"
Early (before booster)	21	High vs low	0		
		Negative vs positive	4 (4^a^)		*BARD1*^a^, *LMBR1*^a^, *TMEM236*^a^, *CCDC158*^a^
Maximum (post booster)	28	High vs low	1 (1^a^)		*DNAH9*^a^
	35	High vs low	0		
Persistence (before slaughtering)	118	High vs low	52 (7^a^)	*TERB2*^a^, *SMPD2*, *ELOVL1*, *NCLN*, *ZNF48*, *CLCN7*, *GSDMD*, *ULK2*, *ZNF668*, *TNFRSF18*, *PTPN18*, *PIM3*, *MARVELD1*, *PHB*, *ETNK2*, *HIST1H4H*, *ZNF787*	*TMTC3*^a^, *ICE2*^a^, *N4BP2*, C*CNC*, *LRIF1*, *AHI1*, *RPAP3*, *STK39*, *ABRAXAS1*, *FAM214A*, *TRAM1*, *ZNF280D*, *AEBP2*, *ZNF350*, *TAOK1*
		Negative vs positive	0		

^a^FDR < 0.05

**Fig. 5:**
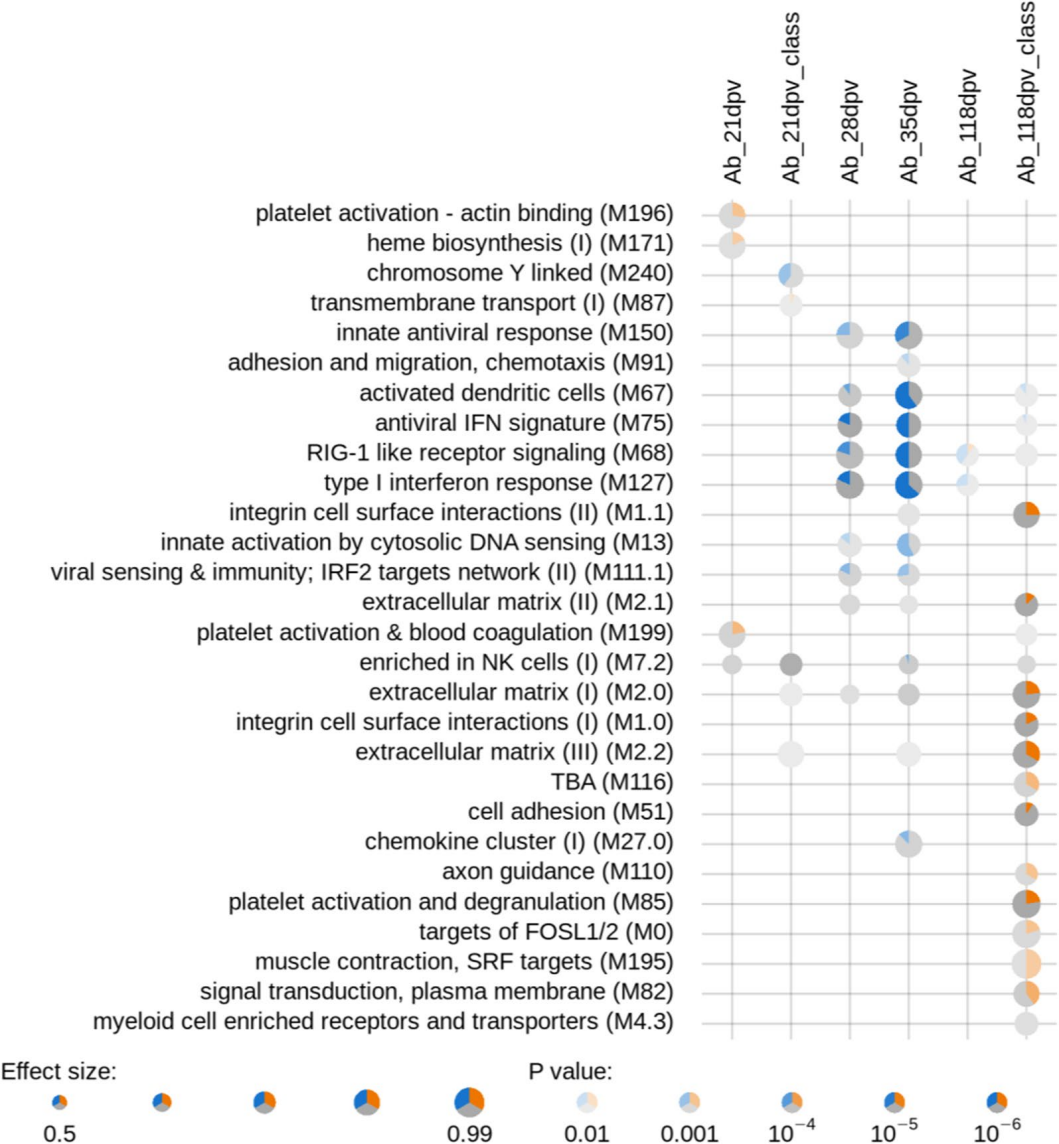
Feature set enrichment analyses (FSEA) of differentially expressed (DE) genes between animals with divergent Ab responses to *M. hyo* vaccination. The CERNO test was operated on lists of genes that were ranked by their absolute log FC of the DE analyses that compare groups of contrasted responders to vaccination at 21, 28, 35, and 118 dpv (high vs low or positive vs negative). BTM significantly enriched in each list (*p* < 0.01 and AUC > 0.75) are represented in rows. BTM are named by their title and ID in parenthesis. The strength of the *p*-value is illustrated by the transparency of color; the effect size (AUC) is illustrated with the plot size. Significantly (*p* < 0.05) up-regulated genes in high or positive responders are colored in blue and downregulated genes are colored in orange; the others are in grey. (TBA = to be annotated)

Since the DE analyses revealed only a few DE genes, functional enrichment analysis was performed using a method that relies on the whole set of analyzed genes that are ranked based on the absolute logFC obtained in the DE analyses. The functions of genes that had a higher expression in the pre-vaccination blood transcriptome of the low responders at 21 dpv were heme biosynthesis (M171) and platelet activation (M196 and M199) (Fig. 5 and Additional file 1: Table S9). Genes of the chromosome Y-linked module (M240) had a higher expression in the pre-vaccination blood transcriptome of positive responders at 21 dpv. For Ab responses at 28 and 35 dpv, genes associated to the same functions had a higher expression in the pre-vaccination blood transcriptome of high responders at both time points. The functions were mainly related to innate antiviral immunity (M13, M68, M75, M111.1, M127, and M150) and to dendritic cells (M67). For the Ab response at 118 dpv, the genes with a higher expression in the pre-vaccination blood transcriptome of the positive responders were annotated to modules related to cell junction and adhesion (M1.0, M1.1, and M51), extracellular matrix (M2.0, M2.1 and M2.2), platelet activation (M85), axon guidance (M110), signal transduction and inflammation (M0 and M82) and myeloid cells (M4.3).

### Blood biomarkers to predict Ab response to *M. hyo* vaccine

**Fig. 6:**
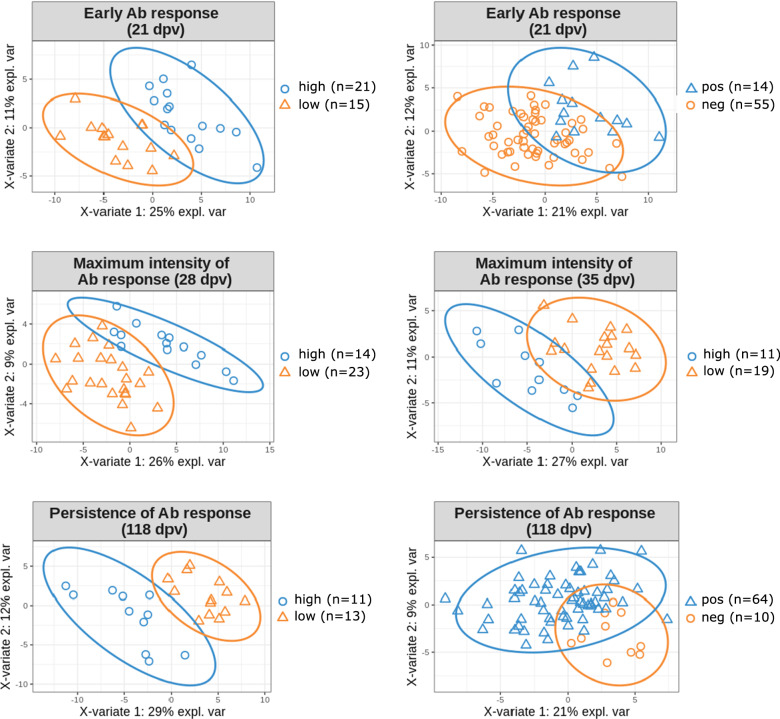
Partial least square-discriminant analysis (PLS-DA) with the 101 predictive genes for *M. hyo* vaccine response. Projection of the groups of contrasted responders to vaccination at 21, 28, 35, and 118 dpv (high vs low or positive vs negative) into the subspace spanned by the first two components after a PLS-DA analysis with the 101 candidate predictive genes. Confidence ellipses for each class are plotted (confidence level set to 95%)

We applied the sPLS-DA method [53] to select predictive genes that can help classify animals as high vs low Ab responders (at 21, 28, 35, and 118 dpv) or positive vs negative Ab responders (at 21 and 118 dpv) (see Additional file 1: Table S10). The detailed contributions of each gene are in Additional file 1: Table S11. Interestingly, the *SH3RF1* gene was a predictor of extreme Ab responders at both 35 and 118 dpv. We defined a set of 101 candidate predictive genes by grouping the genes identified for each time point. PLS-DA were run with the 101-gene set to assess its prediction capacity for classifying animals as high vs low or positive vs negative Ab responders at all time points (Fig. 6 and Additional file 1: Table S11). A good prediction was obtained with the first component (area under the curve (AUC) > 0.86) and a nearly perfect prediction with two or three components (AUC > 0.99), with significant balanced error rates (BER) for Ab responses at all time points (BER ranging from 0.10 to 0.38). Interestingly, among this set of genes, six were in the DE gene list: *NCLN*, ENSSSCG00000032640, *CCDC158*, *TMEM236*, *DNAH9* and *BARD1*. In addition, 10 of these predictive genes have been reported to be under genetic control in an expression GWAS analysis performed on 60-day old pig blood transcriptome [54]: *TXLNB*, *PPP6R3*, *ALMS1*, *PAFAH2*, *SLC39A7*, *CCDC158*, *MASTL*, *DNAH9*, *SENP7*, and *TBC1D12*.

## Discussion

In this study, we characterized the variability of vaccine humoral response to *M. hyo* in pigs. We identified genetic information and pre-vaccination baseline transcriptomic signatures that could predispose to and predict individual *M. hyo* Ab levels induced after vaccination and monitored at different time points: early response after the first injection (21 dpv), maximum intensity response after the booster (28 and 35 dpv), and persistence of Ab response until slaughter (118 dpv).

The mechanisms of the vaccine-induced protection to control *M. hyo* infection in pigs are not yet fully understood. The *M. hyo* vaccine is known to induce both local and systemic immune responses that involve specific Ab production and cellular immunity [5, 55–57]. However, the respective roles of these components of the host immune response on the vaccine-induced protection have not yet been determined and no direct correlation has been reported between the variability of any vaccine response parameter and the protection efficiency after *M. hyo* challenge [5, 55, 56]. Vaccine formulations, routes of administrations, or adjuvants differed between these studies and the number of animals studied were relatively small (less than 10). Studies that include animal challenge with the vaccine pathogen after vaccination on larger populations are needed to establish the correlates of vaccination protection. In our study, we focused on the humoral response following vaccination and found a high inter-individual variability in *M. hyo* Ab levels, influenced by both the host genetics and blood transcriptomics. Thus, we provide a proof of concept that genetic and blood transcriptomic data collected before vaccination are relevant resources to predict vaccine Ab response levels, which could be applied to the correlates of protection that need to be established in future studies.

Ab response levels within an animal were positively correlated with each other across time points, which revealed the absence of an antagonism between Ab response levels at different time points. Selecting for Ab response intensity to vaccination may have an impact on pig production performance due to trade-off issues. Comparisons between vaccinated and non-vaccinated animal groups showed no differences in BW, which highlights that the vaccine has no negative effect on BW at the whole population level. In field conditions where *M. hyo* is circulating, vaccinated pigs have higher growth rates [5, 6], due to the herd protection against the pathogen. In our study, in which no *M. hyo* infection occurred, we observed negative correlations between Ab responses to *M. hyo* vaccination and BW. The high vaccine responders had significantly lower BW and ADG values than the low Ab responders, which suggests a trade-off. Thus, breeding goals should aim at reaching levels of Ab that are sufficient for an efficient protection of animals that persists throughout life, rather than selecting for maximal Ab response capacity. Correlates of protection are still lacking to be able to determine what threshold of Ab level would be sufficient to optimize protection while limiting the decrease in BW. When vaccine response traits are included in breeding programs, a joint assessment of functional trade-offs and economic impacts will be necessary to optimize both vaccine effectiveness and production traits. The balance between sustainability, feasibility and desirability of breeding livestock for disease resistance remains a main issue that needs to be addressed [58].

From the blood transcriptome profiles obtained prior vaccination, we identified differentially expressed genes in animals that showed divergent levels of Ab response to vaccination, especially for persistence of the Ab response. Although these genes were not shared with previously reported gene signatures associated with early response to vaccination in humans or pigs [35, 37, 42], they were enriched in blood cell functions that are biologically relevant to inter-individual variability of Ab response to vaccination. The inflammation, innate antiviral immunity, platelet activation, dendritic cells/antigen presentation, myeloid cells, natural killer cells, and T cell activation functions associated with the Ab response that we have identified in our study have already been reported as gene signatures of vaccination in humans [38, 59], pigs [43], and sheep [39, 40].

The pre-vaccination blood transcriptome predicted *M. hyo* Ab levels at the early and maximum intensity responses, and strikingly at a late time point that corresponded to Ab persistence. Interestingly, the blood cell functions involved in predisposition to vaccination response varied between time points, which suggests that the underlying biological mechanisms involved in the prediction probably differ, and their study needs to be deepened. A high early response was associated with a lower expression in blood of genes that are related to cell cycle and transcription, heme biosynthesis, and platelet activation functions. High responders at the maximum intensity of Ab response showed a higher expression of genes related to dendritic cell, natural killer and T cell activation and antiviral and innate immune response (mainly interferon signature and RIG-1 like sensing), as well as complement activation. The persistence of the Ab response was associated with genes related to cell adhesion, extracellular matrix, platelet activation and monocyte signature. Finally, high Ab responses at nearly all time points were associated with a decreased representation of genes associated to the chromosome Y-specific module (M240) compared to low Ab responses. All these blood cell functions have physiological roles that support their implication in the immune capacity of animals to mount an Ab response to vaccination. The pig Y chromosome harbors genes with regulatory properties that may shape the immune cell transcriptomes [60]. Heme regulates B-cell differentiation and antibody class switch [61, 62] and, in general, an altered CD4 T cell signature can be used as a predictive immune phenotype for low vaccine responsiveness in middle-aged humans [63].

A previous study in pigs did not identify any biomarkers in the blood transcriptome before vaccination for the prediction of *M. hyo* Ab levels measured 10 days after a booster vaccination [42]. It should be noted that, in the same population, the kinome (global cellular kinase activity) revealed prior vaccination candidate biomarkers [32]. In our study, we investigated the early, maximum intensity, and persistence of Ab responses and searched for predictors of the intensity of vaccine response by studying genes that are expressed in pre-vaccination blood. Merging the best predictors of Ab responses at each time point led to a set of 101 genes (see Additional file 1: Table S11) that had an enhanced performance of prediction at all time points compared to single subsets of genes defined at each time point. A sPLS-DA analysis does not necessarily select genes that are of biological relevance to the predicted phenotype. However, six of the 57 genes that were differentially expressed between the high and low responders were found in this set of 101 genes that were predictive of Ab responses. These six genes (*NCLN*, ENSSSCG00000032640, *CCDC158*, *TMEM236*, *DNAH9*, and *BARD1*) were among the best contributors to the prediction. More studies are needed to refine the list of candidate predictors and to validate them in other populations, with different genetic setups and environments of production. Since more and more studies aim at identifying genetic predictors and, more generally, biomarkers of vaccine effectiveness in pigs, it will be essential to analyze whether such predictors apply to different vaccines. Integrating various layers of biological information from the host (genetics, transcripomics, kinomics, etc.) and its gut microbiota is expected to create a breakthrough in our understanding of the determinants of vaccine effectiveness and more globally on immune capacity and animal robustness, with potential applications to improve the sustainability of pig production systems.

In this study, an inter-individual variability of Ab levels in response to *M. hyo* vaccination was reported, with females exhibiting higher levels of Ab than males (uncastrated) at the latest time point. This is consistent with the correlation that we found between a male-specific module (M240) and Ab response that was previously reported in humans for other vaccines [38], and highlights a gender bias in Ab production. Sex differences have been described in immunity to multiple vaccines, including both inactivated and live vaccines in humans, with Ab responses often being higher in females than in males [64, 65]. Thus, sex differences in immune responses to vaccines that could be caused by genetic, hormonal, microbiota, and environmental factors, or a combination of these, should be taken into account in vaccination studies.

In humans, estimates of heritability of Ab responses to several vaccinations ranged from low (< 0.20) to high (> 0.80), depending on the vaccine [23, 66–69]. In pigs, mainly low to moderate heritabilities of specific Ab production have been reported [29, 30]. In a previous study on Large White pigs, we showed that *M. hyo*-specific Ab response after a one-shot injection of vaccine had a large phenotypic variance, with a low heritability of 0.12 [31]. Here, we found heritabilities of *M. hyo*-specific Ab levels ranging from 0.46 to 0.57, with the higher value for the early *M. hyo* Ab levels at 21 dpv. These heritability values might be slightly overestimated, as common environmental effect of birth litter was not included in the final model of analysis due to numerical issues. However, the variance due to common litter effects rarely exceeds 10% of the phenotypic variance, so that heritability values of 0.46 to 0.57 are very likely to be mainly due to additive genetic variation. GWAS revealed two significant peaks for early *M. hyo* Ab levels at 21 dpv, but no associations at other time points. Thus, we confirm the influence of the host genetics on vaccine effectiveness but larger population sizes are needed to go deeper in the analysis of the underlying genetic determinisms, which likely involves numerous genetic loci spread along the genome. In addition, we anticipate that biological relays that are controlled by different genetic determinisms are put in place during the time course of the vaccine response.

The few genes that map to the two GWAS peaks have functional links with immunity in humans. On SSC1, the *DAB2IP* gene encodes the DAB2 interacting protein that functions as a scaffold protein involved in numerous processes including innate immune response, inflammation and cell cycle. A GWAS in humans revealed associations of this gene with aggressive prostate cancer, *DAB2IP* being a candidate tumor suppressor gene [70]. *DAB2IP* is also known to inhibit tumor cell growth in vitro and in vivo, which highlights important properties for anti-cancer therapies [71]. On SSC4, the *ASAP1* gene (A*rfGAP with SH3 domain, ankyrin repeat and PH domain 1*) encodes an ADP-ribosylation factor GTPase-activating protein. *ASAP1* plays a role in regulating the migration of dendritic cells, with a possible impact on predisposition to tuberculosis [72]. Associations of this gene with mean platelet volume and platelet counts have also been reported [73, 74]. The *CYRIB* gene (*CYFIP related Rac1 interactor B*) is related with several pathways, among which response to an elevated concentration of platelet cytosolic Ca2+. GWAS in humans have revealed associations of variants of regulatory elements of *CYRIB* with lymphocyte counts [75]. The *GSDMC* gene (*gasdermin C*) was initially identified as a gene that is preferentially expressed in metastatic melanoma cells [76] and also in the epithelium of the skin and gastrointestinal tract [77], while GWAS in humans revealed associations of variants in *GSDMC* with monocyte counts [78].

Finally, an eGWAS performed on 60-day old pig blood transcriptome revealed that 10 of the 101 genes that were predictive of Ab responses in our study are under genetic control [54]. Deciphering the genetic control of the expression of genes in blood should also help identify the best variant predictors of vaccine responses that could be implemented in breeding programs. Of note, we also recently reported an impact of the fecal microbiota collected before vaccination on vaccine effectiveness in the same population [79], in agreement with findings on another pig population [42]. The accumulation of data on genetic variability and predictors to vaccine responses (transcriptome, kinome, microbiome) should provide new insights to improve vaccination efficacy. From a One-Health perspective, precision livestock production and personalized medicine share the same requirements to better understand the determinants of individual baseline variability that shape immune competence, and to assess if such knowledge will be useful in future breeding practices.

## Conclusions

We showed and thus confirmed a genetic basis for the variability of Ab vaccine response in pigs vaccinated against *M. Hyo*. Heritability of the Ab response at all time points could be estimated and genomic regions associated with the early response (21 dpv) identified. We also identified individual baseline blood transcriptomic signatures prior to vaccination that predicted high or low responses in the tested population. Similar genetic and genomic studies could be applied to the correlates of protection that still need to be established. These results highlight that the variability of baseline genomic expression beyond SNPs may be relevant to predict the variability of vaccine effectiveness and baseline immune competence.

## Supplementary Information

**Additional file 1: Table S1.** Individual data and metadata. **Table S2.** Descriptive statistics of the groups of animals with contrasted Ab response to vaccination against *M. hyo* at different days post-vaccination (dpv) within the total population and the subset for which RNASeq data are available. **Table S3.** Effects of sex, batch, age at weaning and litter on *M. hyo* Ab levels. **Table S4.** Correlations of Ab responses to *M. hyo* vaccination at different days post-vaccination (dpv). **Table S5.** Effects of sex, batch, age at weaning, BW at 0 dpv, vaccination and litter on BW at 40 and 118 dpv and ADG from 0 to 40 and 40 to 118 dpv. **Table S6.** Genetic parameters of Ab responses to *M. hyo* vaccination at different days post-vaccination (dpv). **Table S7.** Suggestive (FDR < 0.1) and significant (FDR < 0.05) SNPs associated with the early *M. hyo* Ab levels at 21 dpv (sera diluted 1/4). **Table S8.** FSEA of transcript overlap between WGCNA modules and BTM. **Table S9.** FSEA of differentially expressed genes between groups of animals with contrasted Ab responses to *M. hyo* vaccination. **Table S10.** Determination of the number of genes (maximum 25) necessary to reach an optimized prediction of extreme Ab responders to *M. hyo* vaccination by sPLS-DA. **Table S11.** Contribution to the sPLS-DA analysis of the different phenotypes of the set of 101 genes predictive of extreme Ab responders to *M. hyo* vaccination. **Table S12.** PLS-DA with the set of 101 genes predictive of extreme Ab responders to *M. hyo* vaccination.

## Data Availability

The blood transcriptome RNA-Seq datasets generated and analysed during the current study are available in the SRA repository with accession number PRJNA661433. All other data generated or analysed during this study are included in this published article and its additional files.
